# The ovulation trigger–OPU time interval of different ovarian protocols in ART: a retrospective study

**DOI:** 10.1007/s00404-020-05568-5

**Published:** 2020-06-03

**Authors:** Xi Shen, Hui Long, Wenya Guo, Yating Xie, Hongyuan Gao, Jie Zhang, Yun Wang, Qifeng Lyu, Yanping Kuang, Li Wang

**Affiliations:** Department of Assisted Reproduction, Shanghai Ninth People’s Hospital Affiliated to Shanghai Jiao Tong University School of Medicine, Shanghai, 200011 People’s Republic of China

**Keywords:** Ovulation trigger–OPU interval time, Retrieved oocyte rate, Mature oocyte rate, Controlled ovarian hyperstimulation protocol

## Abstract

**Purpose:**

To explore the trends of oocyte and pregnancy outcomes over the ovulation trigger–OPU (oocyte pickup) time interval in four mainly used COH protocols.

**Methods:**

This retrospective study was conducted between January 2013 and July 2018. The IVF/ICSI cycles of the patients with normal ovarian reserve were included. The number of total patients was 4673, which consisted of long agonist protocol (*n* = 819), short agonist protocol (*n* = 1703), mild stimulation protocol (*n* = 1627), and GnRH antagonist protocol (*n* = 524). The primary outcome was mature oocyte rate.

**Results:**

The ovulation trigger–OPU time interval and COH protocol were related to cycles with > 80% MII oocytes. Four protocols showed apparently different trends of retrieved oocyte rate and mature oocyte rate over the ovulation trigger–OPU time interval, and the long agonist protocol had the most delayed time interval than other three COH protocols in retrieving more than 60% oocytes (35.4–39.6 h vs. 34.6–38.6 h vs. 32.5–37.5 h vs. 33.8–37.7 h) and getting more than 80% mature oocytes (35.0–39.7 h vs. 36.0–37.7 h vs. 34.1–35.5 h vs. 34.5–36.3 h). And the adjusted odds ratio (OR) of the cumulative live birth rate (CLBR) (OR 1.360, 95% Confidence Interval (CI) 1.156–1.549, *P* < 0.05) significantly increased with the trigger–OPU time interval in the long agonist protocol.

**Conclusions:**

For getting more and mature oocytes, the ovulation trigger–OPU time intervals should be gradually prolonged from the mild stimulation protocol, the GnRH antagonist protocol, and the short protocol to the long agonist protocol. And the prolonged ovulation trigger–OPU time interval in the long agonist protocol brings higher live birth rate (LBR) and CLBR.

**Electronic supplementary material:**

The online version of this article (10.1007/s00404-020-05568-5) contains supplementary material, which is available to authorized users.

## Introduction

The time interval from trigger to oocyte pickup (OPU) is vital, which consists of the luteinization start, the cumulus cell expansion, and the reduction division of the oocyte resumption [1]. In most in vitro fertilization (IVF) cycles, the commonly practiced interval was 32–36 h, which was derived from the studies on patients who used Clomiphene Citrate (CC) and/or human menopausal gonadotropin (hMG) for ovulation induction [2, 3].

The clinical results of ART vary along with the time interval between oocyte maturation trigger and aspiration. Some studies have found that longer OPU time did not lead to more mature oocytes or better clinical results [4–8], whereas other studies have found that longer OPU time may produce more mature oocytes [1, 9, 10], higher fertilization rate [11], better blastocyst development [12], more good-quality embryos, and higher ongoing pregnant rate [13]. There were also some studies which showed prolonging the interval between human chorionic gonadotropin (hCG) priming and oocyte retrieval could only increase the proportion of MII oocytes, rather than the pregnancy rates [14, 15]. Therefore, most research focused on whether doctors should delay the OPU time in the specific controlled ovarian hyperstimulation (COH) protocol, such as the gonadotropin-releasing hormone analog (GnRHa) protocol.

A few studies researched the individual optimal ovulation trigger–OPU interval in the different COH protocols, which probably exist and need to be paid attention to, especially when COH protocols getting diverse. For example, GnRHa combined with hMG is different from the CC or hMG stimulation protocols; the former one needs longer oocyte OPU hours, usually 34–38 h [5, 9]. With current knowledge, there was only one meeting abstract firstly reporting the individual optimal trigger–OPU intervals of different COH protocols: 35–36 h in long protocol, 35–37 h in flare-up protocol, and 36–37 h in antagonist protocol [16].

Therefore, this retrospective analysis with a huge number of patients who used different COH protocols first explored the trends of oocyte and pregnancy outcomes over the ovulation trigger–OPU time interval in four mainly used COH protocols, to give a reference in arranging the ovulation trigger–OPU time intervals within patients using different COH protocols on the same day.

## Materials and methods

### Study setting and patients

This retrospective study was conducted between January 2013 and July 2018 in the Department of Assisted Reproduction of the Ninth People’s Hospital affiliated to Shanghai Jiao Tong University School of Medicine. The following inclusion criteria were applied: women aged less than 40 years; basal follicle stimulating Hormone (FSH) < 10 m IU/ml; antral follicle count (AFC) > 5. The infertility reasons were tube factors, male factors, combined factors, and other factors (repeated intrauterine insemination failures and unknown reasons). Study following exclusion criteria was applied: (1) patients with endometriosis and polycystic ovarian syndrome; (2) E_2_ level on the day after trigger decrease more than 10% than the trigger day, which could induce the higher possibility of spontaneous premature ovulation and lower oocyte retrieval rate [17], the detailed number was as follows: 36 patients (4.21%) in long agonist protocol; 49 patients (2.80%) in short agonist protocol; 55 patients (3.27%) in mild stimulation protocol; 23 patients (4.20%) in GnRH antagonist protocol; (3) receipt of hormone treatments within the previous 3-month period and (4) any contraindications to ovarian stimulation treatment.

### Ovarian stimulation protocols

The four COH regimens are described briefly as follows. In the long agonist protocol, the long-acting gonadotrophin-releasing agonist (leuprorelin acetate, 3.75 mg, Lizhu Pharmaceutical Trading Co., China) was administered on cycle days 2–5. If downregulation was quantified (E_2_ < 50 pg/ml) after 35 days, then hMG (150–225 IU/day) was administered until trigger day. In short agonist protocol, patients were administered 0.1 mg of short-acting gonadotrophin-releasing agonist (triptorelin, 0.1 mg, Ferring Pharmaceuticals, China) daily beginning on MC2 and 150–225 IU of hMG daily beginning on cycle day 3. The mild ovarian stimulation protocols were flexible and consisted of letrozole (2.5 mg, Jiangsu Hengrui Medicine Co. Ltd, Lianyungang, China), clomiphene (50 mg/day; Codal Synto Limited, Limassol, France) and hMG (75–150 IU) [18]. In GnRH-antagonist protocol, hMG (150-225 IU/day) was administered and the GnRH antagonist, cetrorelix (Pierre Fabre Medicament Production-Aquitaine Pharm International, France), was next administered daily by s.c. injection (0.125–0.25 mg/day) when the diameter of one follicle reach 14 mm [19]. The doses were adjusted according to the transvaginal ultrasound examination. For all protocols, when three dominant follicles reached at least 18 mm or one dominant follicle reached at least 20 mm in diameter, the final oocyte maturation was triggered using Triptorelin (0.1 mg, Ferring Pharmaceuticals) and/or hCG (5000 IU; Lizhu Pharmaceutical Trading Co., China) in mild stimulation protocol as well as GnRH antagonist protocol, while triggered by hCG (5000 IU) in short agonist protocol and long agonist protocol.

### Oocyte retrieval operation, insemination, and embryo culture

Oocyte aspirations were conducted by one skillful physician in our center. All follicles more than 10 mm would be aspirated on the retrieved day. Aspirated oocytes were transferred to the embryology laboratory in Modified HTF Medium (Irvine Scientific, USA), and then, they were transferred to the culture medium. Fertilization was carried out via either IVF or intracytoplasmic sperm injection (ICSI), depending on the semen parameters of the patient’s husband [20]. For IVF, fertilization was performed after about 4–6 h and the maturity was examined on the next day of OPU. In the situation of ICSI, oocytes were denuded and examined for maturity after 2–3 h preincubation. The immature oocytes were examined again after 2 h and all the mature oocytes were injected [21, 22]. According to the Cummins’ standard, the third-day good-quality embryos were defined as grade 1 and grade 2 embryos with at least eight cells [23, 24]. Non-top-quality embryos were placed in extended culture until they reached the blastocyst stage. The good morphology blastocysts and the cleavage good-quality embryos were defined as viable embryos, which could be used for fresh or frozen embryo transfer (FET).

### Statistical analysis

The primary outcome was mature oocyte rate. The second outcomes were oocyte retrieval rate, live birth rate, and cumulative live birth rate. The time interval of oocyte aspiration was defined as the midpoint of the start time (patient transferred to the operating table) and the end time (patient removed from the operating table). The following definitions were used: oocyte retrieval rate = the number of cumulus–oocyte complex (COC) retrieved/the number of follicles ≥ 10 mm present on the retrieval day; mature oocyte rate = the number of mature oocyte/the number of oocytes retrieved. Live births were defined as at least 22 gestational weeks or at least 500 g. Cumulative live birth rate (CLBR) was defined as the number of deliveries with at least one live birth resulting from one aspirated ART cycle [25].

Statistical analyses were carried out using SPSS software (version 22, SPSS Inc., Chicago, IL, USA). Variables were expressed as the means ± SD, which were tested with one-way ANOVA. Qualitative data are presented as percentages and were tested with the Chi-squared test or Fisher’s exact test when appropriate. A logistic regression model was performed to quantify the related factors of cycles with more than 80% mature oocytes and the odds ratio of pregnancy-related outcomes. The adjusted odds ratio was calculated using the significant confounding factors selected from age, infertility duration, BMI, fertilization methods, AFC, and hMG doses. Additionally, locally estimated scatterplot smoothing (Loess) was adjusted with the R software (R for windows, 3.4.4 version) to relate the percentage of oocyte retrieval and oocyte maturity trend over the lag trigger–OPU time interval. The time interval for getting more than 60% oocyte retrieval rate and 80% mature oocyte rate was defined as the junction of lower confidence interval limit [26] and reference line. Statistical significance was defined as a comparison resulting in *P* < 0.05.

## Results

### Related factors of cycles with more than 80% MII oocytes

A logistic regression analysis was performed to analyze the related factors of cycles with more than 80% MII oocytes (Table 1). The data illustrated that the factors of cycles with > 80% MII oocytes were not significantly related to age, BMI, and infertility duration (*P* > 0.05), nor with the oocyte trigger method [GnRH agonist as reference (ref.); hCG group: odds ratio (OR) 1.234, 95% confidence interval (CI) 0.803–1.896, *P* > 0.05; dual trigger group: OR 1.082, 95% CI 0.719–1.629, *P* > 0.05]. However, the antral follicle number (OR 0.981, 95% CI 0.966–0.996, *P* = 0.017) was a negative correlation factor and gonadotropin days (OR 1.038, 95% CI 1.010–1.066 *P* = 0.007) were a positive factor of cycles more than 80% mature oocytes.

**Table 1:** Logistic regression analysis of factors associated with cycles with > 80% mature oocytes

Parameter		OR (95% Cl)	*P* value
Age (years)		1.009 (0.990–1.028)	0.367
Antral follicle count (n)		0.981 (0.966–0.996)	0.017
Infertility duration (year)		0.984 (0.953–1.016)	0.312
Body mass index (kg/m^2^)		0.977 (0.951–1.004)	0.092
Gonadotropin days (day)		1.038 (1.010–1.066)	0.007
Trigger–OPU time interval (h)	< 35.00	Reference	
	35.01–36.00	1.124 (0.908–1.393)	0.283
	36.01–37.00	1.309 (1.028–1.666)	0.029
	37.01–38.00	1.130 (0.863–1.479)	0.375
	> 38.01	1.265 (0.908–1.761)	0.165
Ovulation stimulation protocol	Long agonist protocol	Reference	
	Short agonist protocol	0.612 (0.479–0.782)	< 0.001
	Mild stimulation protocol	1.002 (0.605–1.659)	0.995
	GnRH antagonist protocol	0.815 (0.542–1.225)	0.326
Ovulation trigger method	GnRH agonist	Reference	
	hCG	1.234 (0.803–1.896)	0.338
	Dual trigger (GnRH agonist + hCG)	1.082 (0.719–1.629)	0.705

Notably, there were another two significant factors related to cycles with > 80% MII oocytes. One was the oocyte aspiration interval time after triggering (< 35 h group as ref.; 35.01–36.00 h group: OR 1.124, 95% CI 0.908–1.393, *P* > 0.05; 36.01–37.00 h group: OR 1.309, 95% CI 1.028–1.666, *P* = 0.029; 37.01–38.00 group: OR 1.130, 95% CI 0.863–1.479, *P* > 0.05; > 38.01 h group: OR 1.265, 95% CI 0.908–1.761, *P* > 0.05). The other factor was the different COH protocols (long agonist protocol group as ref.; Short agonist protocol group: OR 0.612, 95% Cl 0.479–0.782, *P* < 0.001; Mild stimulation protocol group: OR 1.002, 95% Cl 0.605–1.659, *P* > 0.05; and GnRH antagonist protocol group: OR 0.815, 95% Cl 0.542–1.225, *P* > 0.05). These results indicated that the percentage of oocyte maturity was related to ovulation trigger–OPU time interval and COH protocol, which supported us to continue to explore the individual ovulation trigger–OPU time interval of four COH protocols.

### Four COH protocols showed different trends of oocyte retrieval rate along with their ovulation trigger–OPU time intervals

**Fig. 1:**
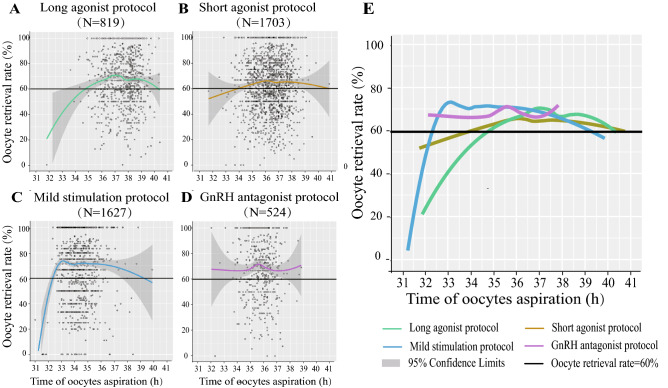
Scatterplot of four COH protocols demonstrating the oocyte retrieval as a function of lag time from ovulation trigger to ovum aspiration in IVF/ICSI, featuring loess trend with the 95% confidence interval highlighted with gray color. **a**–**d** Oocyte retrieval rate separately in the long agonist protocol, short agonist protocol, mild stimulation protocol, and GnRH antagonist protocol. **e** Merged image of **a**–**d**

Figure 1 showed a scatterplot for oocyte retrieval rate on the *y*-axis vs. the lag time on the *x*-axis in IVF/ICSI within four COH protocols. Baseline characteristics of four COH protocols were first analyzed and comparable in the age, basal FSH, AFC, infertility duration, primary infertility, infertility reasons, BMI, and insemination methods (IVF, ICSI, IVF + ICSI) (*P* > 0.05) (Supplement Table 1).

However, the inherent characters of four COH protocols showed the apparent difference, such as gonadotropin (Gn) doses, E_2_ level on the trigger day, and E_2_ level on the day after trigger day, which were also the key important regulators in ovulation. Therefore, four COH protocols had apparently different trends of oocyte retrieval rate along with their ovulation trigger–OPU time intervals in IVF/ ICSI (Fig. 1a–d) and ICSI (Sup Fig. 1 A1–D1). The oocyte retrieval rate was almost higher than 60% in the long agonist protocol (Fig. 1a, 35.4–39.6 h), short agonist protocol (Fig. 1b, 34.6–38.6 h), mild stimulation protocol (Fig. 1c, 32.5–37.5 h), and GnRH antagonist protocol (Fig. 1d, 33.8–37.7 h). The ovulation trigger–OPU time interval was most delayed in the long agonist protocol (Fig. 1e and Sup Fig. 1E1).

### The trends of mature oocyte rate were also different over the ovulation trigger–OPU time intervals of four COH protocols

**Fig. 2:**
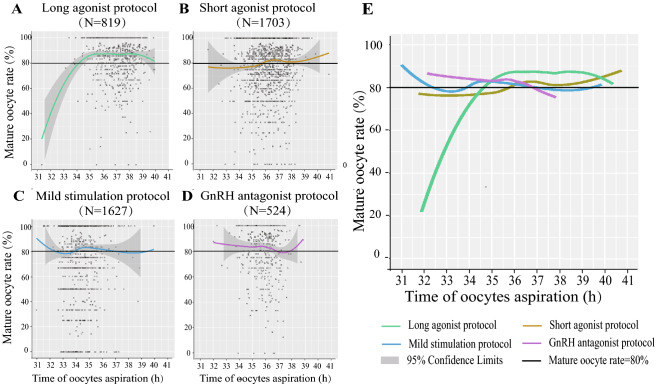
Scatterplot of four COH protocols demonstrating the mature oocyte rate as a function of lag time from ovulation trigger to ovum aspiration in IVF/ICSI, featuring loess trend with the 95% confidence interval highlighted with gray color. **a**–**d** Mature oocyte rate separately in the long agonist protocol, short agonist protocol, mild stimulation protocol, and GnRH antagonist protocol. **e** Merged image of **a**–**d**

Except for the oocyte retrieval rate, four COH protocols also had different trends of mature oocyte rate over the lag time from ovulation trigger to ovum aspiration in IVF/ICSI (Fig. 2a–d) and ICSI (Sup Fig. 1 A2–D2). 80% of mature oocyte rate was defined as a reference line, being not easy to achieve in the clinic. Seeing from the merged data (Fig. 2e and Sup Fig. 1 E2), the gradual delayed tendency of ovulation trigger–OPU time intervals from the mild stimulation protocol (34.1–35.5 h in IVF/ICSI; 32.8–35.3 h in ICSI), the GnRH antagonist protocol (34.5–36.3 h in IVF/ICSI; None in ICSI), and the short protocol (36.0–37.7 h in IVF/ICSI; 35.1–37.2 h in ICSI) to the long agonist protocol (35.0–39.7 h in IVF/ICSI; 35.5–37.5 h in ICSI), was also existed in the mature oocyte rate.

### The summary of pregnancy outcomes over the ovulation trigger–OPU time intervals in four COH protocols

Unlike the oocyte retrieval rate and mature oocyte rate, not all the pregnancy outcomes are closely related to the ovulation trigger -OPU time intervals in four COH protocols (Table 2). After adjusting confounders, comparing with the implantation rate and clinical pregnancy rate, only the adjusted live birth rate per transfer (OR 1.196, 95% CI 1.045–1.368, *P* < 0.05) and the CLBR (OR 1.360, 95% CI 1.156–1.549, *P* < 0.05) had positive relations with their trigger–OPU time intervals of long agonist protocol in four COH protocols.

**Table 2:** The odds ratio of pregnancy outcomes with trigger–OPU time interval

	Crude OR (95% CI)	*P*	Adjusted OR (95% CI)	*P*
Clinical pregnancy rate				
Long agonist protocol	1.081 (0.956–1.223)	0.213	1.129 (0.976–1.305)	0.102
Short agonist protocol	0.955 (0.876–1.041)	0.297	0.971 (0.883–1.067)	0.535
Mild stimulation protocol	1.085 (0.963–1.223)	0.181	1.096 (0.967–1.242)	0.150
GnRH antagonist protocol	0.806 (0.656–0.990)	0.040	0.860 (0.685–1.063)	0.163
Implantation rate				
Long agonist protocol	1.045 (0.943–1.157)	0.405	1.083 (0.960–1.221)	0.194
Short agonist protocol	0.964 (0.897–1.035)	0.311	0.951 (0.885–1.021)	0.167
Mild stimulation protocol	1.065 (0.966–1.175)	0.205	0.850 (0.707–1.021)	0.082
GnRH antagonist protocol	0.816 (0.689–0.968)	0.019	0.867 (0.728–1.033)	0.111
Live birth rate per transfer				
Long agonist protocol	1.181 (1.033–1.350)	0.015	1.196 (1.045–1.368)	0.009
Short agonist protocol	0.977 (0.892–1.071)	0.622	1.013 (0.988–1.038)	0.318
Mild stimulation protocol	1.091 (0.966–1.232)	0.162	1.034 (0.901–1.187)	0.631
GnRH antagonist protocol	0.878 (0.709–1.086)	0.230	0.940 (0.754–1.171)	0.580
Cumulative live birth rate				
Long agonist protocol	1.311 (1.143–1.502)	0.000	1.360 (1.156–1.549)	0.020
Short agonist protocol	1.033 (0.942–1.132)	0.495	0.947 (0.816–1.098)	0.471
Mild stimulation protocol	1.215 (1.086–1.359)	0.001	1.088 (0.956–1.237)	0.200
GnRH antagonist protocol	0.794 (0.645–0.978)	0.030	0.859 (0.695–1.063)	0.163

Table 3 shows the overview of ovulation trigger–OPU time intervals in four COH protocols. This timing advance tendency of mild stimulation protocol and GnRH antagonist protocol was seen from the 80% concentrated time intervals, which was further reflected in the ovulation trigger–OPU time period with more than 60% oocyte retrieval rate and more than 80% mature oocyte rate. And the long agonist protocol had the most delayed ovulation trigger–OPU time interval with > 60% oocyte retrieval rate (35.4–39.6 h, 4.2 h) and > 80% mature oocyte rate (35.0–39.7 h, 4.7 h).

**Table 3:** Description of trigger–OPU time period in different COH protocols

	Total trigger–OPU time period (h)	Trigger–OPU time period (concentrated in 10th–90th, h)	Trigger–OPU time period (> 60% oocyte retrieval rate, h)	Trigger–OPU time period (> 80% mature oocyte rate, h)	Relationship with LBR and CLBR
Long agonist protocol	31.8–40.4 (8.6)	36.0–39.0 (3.0)	35.4–39.6 (4.2)	35.0–39.7 (4.7)	Positive
Short agonist protocol	31.7–40.9 (9.2)	35.0–38.0 (3.0)	34.6–38.6 (4.0)	36.0–37.7 (1.7)	None
Mild stimulation protocol	30.4–39.9 (9.5)	33.0–35.5 (2.5)	32.5–37.5 (5.0)	34.1–35.5 (1.4)	None
GnRH antagonist protocol	32.0–38.8 (6.8)	34.5–37.0 (2.5)	33.8–37.7 (3.9)	34.5–36.3 (1.8)	None

## Discussion

This study first explored that the ovulation trigger–OPU time interval and COH protocol are two main factors relating to cycles more than 80% MII oocytes. To retrieve more than 60% oocytes and get more than 80% mature oocytes, the ovulation trigger–OPU time intervals of four COH cycles were apparently different and had a gradual delayed tendency among the mild stimulation protocol, GnRH antagonist protocol, short agonist protocol, and the long agonist protocol. Moreover, the ovulation trigger–OPU time interval was positively corelated with the cumulative live birth rate in the long agonist protocol, rather than other three COH protocols.

### Strengths and limitations

This research first overviews that four COH protocols which are usually used in the clinic at present have their individual explicit features of ovulation trigger–OPU time intervals, not only considering from the oocytes performance such as oocyte retrieval rate and mature oocyte rate, also from pregnancy outcomes such as LBR and CLBR. Actually, there is a lack of specialized and systematic research on this aspect. Furthermore, former studies usually divided their trigger–OPU time into several subgroups and focused on the diversity between the shorter period and longer period [5, 9]. However, our study pays attention to the effect of continuous time interval on clinical outcomes, which could take advantage of more useful and intuitive information.

Our study also has some limitations. This is a retrospective study without randomization. Second, we analyzed the relationship of ovulation trigger–OPU time interval with the pregnancy outcomes in four COH protocols. However, the transfer strategy of four COH protocols are different: the GnRH antagonist and long protocols are matched with both fresh embryo transfer (ET) and FET, while the short protocol and the mild stimulation protocol used FET, and sometimes, ET would be chosen. Therefore, we calculated not only the live birth rate, but also the cumulative live birth rate (CLBR), which included the ET and subsequent FET cycles. However, the randomized-controlled research combined with the same transfer method is need to explore this question in the future.

### Comparisons of results between ours and previous studies

Some studies considered that mature oocyte rate had no significant relationship with basic parameters [14]. In our research, we found that there were some basic parameters associating with mature oocyte rate. Interestingly, AFC is negatively relative with mature oocyte rate. The possible reason may be that although higher AFC could lead to more retrieved oocytes, they also contributed to higher proportion of small immature oocytes on the retrieval day, which may result in lower oocyte maturity rate. Because Anti-Mullerian Hormone (AMH), which is another indicator of ovarian reserves and strong positively with AFC, also had higher correlation coefficients with immature oocytes than mature oocytes [27, 28].

Previous research mostly explored the optimal ovulation trigger–OPU time interval in one specific COH protocol. The early research found the proper trigger–OPU interval in patients stimulated with clomiphene citrate (CC) or hMG was 32–36 h [2, 29, 30]. After that, lots of research explored whether oocytes should be retrieved earlier or later in the gonadotropin-releasing hormone analog (GnRHa) combined with hMG protocol [1, 5, 9, 13, 14, 31, 32]. For the different GnRHa protocols which may bring the distinct extent of pituitary suppression and need diverse in vivo maturation time before oocyte retrieving, the only related research is one meeting abstract [16], which found the long protocol, flare-up, and antagonist protocols had their discrepant optimal time for oocyte retrieval in terms of egg maturation, fertilization, implantation, and clinical pregnancy rates. Our research further illustrated the diversity of trends in oocyte and pregnancy outcomes with trigger–OPU time interval in four COH protocols mainly used at present.

We explored the trends of oocyte retrieval rate and mature oocyte rate along with the ovulation trigger–OPU time intervals mainly in IVF/ISCI, but also in ICSI. The oocyte maturity in ICSI is an important and necessary supplement to evaluate the features of ovulation trigger–OPU time intervals in those COH cycles. The ovulation trigger–OPU time periods were also apparently different in the four COH protocols and showed the delaying tendency from the mild stimulation protocol, GnRH antagonist protocol to the short and long agonist protocol, same as in the IVF/ICSI. Some groups had limited sample sizes such as GnRH antagonist protocol with ICSI (*N* = 157, Supplement Fig. 1 D2 and E2), which may lead to some biases and the worst outcome in long time interval. A more accurate evaluation of oocyte performance would be performed with larger sample size.

### Possible mechanisms of the individual optimal ovulation trigger–OPU time intervals of different COH protocols

As the classic protocols used all over the world, the GnRH analogs had lots of types. The short agonist protocol, long agonist protocol, and GnRH antagonist protocol all suppress E_2_-positive feedback and the preovulatory GnRH/LH surge in the pituitary, but the suppression time period is different. The GnRH antagonist is administrated after the oocytes growing up, while the long agonists are usually administrated 1 month before ovarian hyperstimulation and short agonists are initially injected at the same time with hMG [33], which may bring the different extent of pituitary suppression. Therefore, the ovulation trigger–OPU time intervals for getting over 80% mature oocytes seem having the gradual prolonged tendency among the GnRH antagonist protocol (34.5–36.3 h), the short protocol (36.0–37.7 h), and the long agonist protocol (35.0–39.7 h).

Consistent with our clinical experience, the long agonist protocol has the most delayed ovulation trigger–OPU time interval. And the explanation may be that the lowest LH level on trigger day in long agonist protocol (Supplement Table 1). When the same doses of hCG act on LH receptors, the lowest LH level may need more time to finish the process of cumulus cell oocyte complex (COC) expansion. Moreover, the lower follicular LH level may result in higher sensitivity of LH receptors, which leads to higher concentration of amphiregulin and promotes oocyte maturation [34].

Furthermore, the mild stimulation protocol had a relatively earlier trend in retrieving oocytes, owing to its specific inherent characters in superovulation. The usage of CC has the antiestrogenic effect, which may suppress premature LH surge. However, this inhibition function of CC is less effective than GnRH analog, which could also be seen in the higher cancelation rate [35], so we usually pick up the oocytes early to avoid the preovulation. Our study also indicates that the highest oocyte retrieval rate was observed between 32 and 33 h; however, the mature oocyte rate was obviously higher in later time period (Figs. 1c, 2c). This may explain why we usually retrieve the oocytes early enough to avoid preovulation in mild stimulation protocol, but which may impair the cytoplasmic maturation and oocyte development competence and bring this unsatisfactory oocyte maturation.

Last but not least, not only LBR per transfer but also CLBR is concerned due to FET cycles. According to Deng et al. [10], the extended time interval between hCG administration and oocyte retrieval might increase the cumulative live birth rate of embryos from a cohort of oocytes retrieved during one cycle when adopting FET strategy. Longer ovulation trigger–OPU time interval could bring out a higher trend in oocyte retrieval rate and mature oocyte rate, which would lead to more mature oocytes in later time period and more subsequent transplant FET cycles, thus resulting in higher CLBR. However, no significant increase was observed in the other three protocols. Our study shows that the trigger–OPU time interval related more closely with oocyte outcomes than pregnancy outcomes. This may be due to that the trigger–OPU interval could partly influence nuclear and cytoplasmic maturity of oocytes, but more factors would take part in the later process of embryonic development and implantation [36].

## Conclusion

The ovulation trigger–OPU time interval and COH protocol are two main factors relating to cycles more than 80% mature oocytes. The ovulation trigger–OPU intervals for retrieving > 60% oocytes and getting > 80% maturity are apparently different in four COH protocols, and had a gradual prolonged tendency from the mild stimulation protocol, the GnRH antagonist protocol, and the short protocol to the long agonist protocol. Moreover, the prolonged ovulation trigger–OPU intervals had a significantly positive relation with a live birth rate per transfer and cumulative live birth rate only in the long agonist protocol.

## Electronic supplementary material

Below is the link to the electronic supplementary material.

Supplementary file1 (DOCX 18 kb)

**Supplement Figure 1** Scatterplot of four COH protocols demonstrating the oocyte retrieval and mature oocyte rate as a function of lag time from ovulation trigger to ovum aspiration in ICSI, featuring loess trend with the 95% confidence interval highlighted with grey color. A1, B1, C1, D1 represent the oocyte retrieval rate separately in the long agonist protocol, short agonist protocol, mild stimulation protocol and GnRH antagonist protocol. A2, B2, C2, D2 represent the mature oocyte rate in the four protocols described above. E1 and E2 show the merged image of A1-D1 and A2-D2, respectively (EPS 31157 kb)
