# Supplementary material for: The ovulation trigger–OPU time interval of different ovarian protocols in ART: a retrospective study
Source: Arch Gynecol Obstet. 2020 Jun 3;302(2):519–27. doi: 10.1007/s00404-020-05568-5 (PMC7321905; doi:10.1007/s00404-020-05568-5)
Supplement: Supplementary file 1 — Supplementary file1 (DOCX 18 kb) [file 404_2020_5568_MOESM1_ESM.docx]

| Supplement Table1 Basic features of patients with four different protocols | | | | | |
| --- | --- | --- | --- | --- | --- |
| Pamameter | Long protocol | Short  protocol | Mild stimulation protocol | GnRH antagonist protocol | P value |
| No. of patients (n) | 819 | 1703 | 1627 | 524 |  |
| Age (year) | 31.05±3.32 | 31.20±3.55 | 31.04±3.78 | 31.07±3.74 | 0.583 |
| Basal FSH (mIU/ml) | 5.46±1.57 | 5.55±1.25 | 5.51±1.26 | 5.61±1.32 | 0.230 |
| AFC (n) | 12.10±4.25 | 12.08±4.27 | 11.93±4.45 | 12.37±4.24 | 0.252 |
| BMI (kg/m^2^) | 21.82±3.05 | 21.84±2.94 | 22.26±5.58 | 22.01±3.06 | 0.072 |
| Infertility duration (year) | 3.06±2.39 | 3.15±1.99 | 3.13±2.00 | 2.95±2.07 | 0.229 |
| Primary infertility n(%) | 445(54.33) | 918(53.81) | 877(53.90) | 297(56.70) | 0.704 |
| Infertility reasons n(%) |  |  |  |  | 0.793 |
| Tube factors | 510(62.27) | 1079(63.36) | 1014(62.32) | 322(61.45) |  |
| Male factors | 117(14.29) | 217(12.74) | 208(12.78) | 76(14.50) |  |
| Combined | 17(2.08) | 38(2.23) | 36(2.21) | 6(1.15) |  |
| Others | 175(21.37) | 369(21.67) | 369(22.68) | 120(22.90) |  |
| Gn dose (IU) | 2639.26±936.56^a^ | 1689.31±774.65^b^ | 633.68±409.81 ^c^ | 2016.65±872.45 ^d^ | 0.000 |
| LH level on the trigger day (mIU/ml) | 0.82±0.94 ^a^ | 4.38±2.21 ^b^ | 6.66±2.91 ^c^ | 1.99±1.64 ^d^ | 0.000 |
| E_2_ level on the trigger day (pg/ml) | 2823.61±1450.71^a^ | 3290.81±1468.19 ^b^ | 929.92±761.80 ^c^ | 2673.08±1510.80 ^a^ | 0.000 |
| Insemination methods n(%) |  |  |  |  | 0.234 |
| IVF | 505(60.0) | 983(59.0) | 1005(63.1) | 317(61.6) |  |
| ICSI | 266(31.6) | 531(31.9) | 444(27.9) | 157(30.5) |  |
| IVF+ICSI | 71(8.4) | 151(9.1) | 143(9.0) | 41(8.0) |  |

Note：Different alphabet a,b,c,d means significant difference (Bonferroni method) between groups
